# Mirtazapine treatment of diabetic gastroparesis as a novel method to reduce tube-feed residual: a case report

**DOI:** 10.1186/1752-1947-7-38

**Published:** 2013-02-06

**Authors:** Janelle Y Gooden, Paul Y Takahashi

**Affiliations:** 1 Mayo Clinic, 200 First Street SW, Rochester, MN, 55905, USA

**Keywords:** Diabetes, Gastroparesis, Management, Mirtazapine, Recalcitrant

## Abstract

**Introduction:**

Gastroparesis is a common motility disorder that is characterized by delayed gastric emptying in the absence of mechanical obstruction. Diabetes, along with other neuromuscular and infiltrating disorders, can predispose individuals to an increased risk of developing gastroparesis. Gastroparesis can be easily diagnosed through gastric emptying studies but is usually difficult to successfully treat. Therapy usually begins with pro-kinetic and anti-emetic agents.

**Case presentation:**

Our patient was an 87-year-old African-American woman who was a nursing home resident, with a history of diabetes mellitus type 2 and subarachnoid hemorrhage leading to aphasia, hemiplegia, seizures and dysphagia requiring percutaneous gastric feeds. While at the nursing home, she had recurrent aspiration pneumonia and large tube-feed residuals consistent with a diagnosis of underlying gastroparesis. Her management included metoclopramide and reduced tube-feeding rates, which improved her symptoms. However, within months the aspiration and increased residuals returned. After trials of different medication therapies without success, she started mirtazapine and her residual volume and aspirations decreased with a dose of 15mg nightly.

**Conclusion:**

In patients with gastroparesis recalcitrant to first line therapies such as metoclopramide, off-label use of mirtazapine may provide adequate non-invasive management of gastroparetic symptoms.

## Introduction

Gastroparesis is a motility disorder involving delayed gastric emptying with no evidence of physical obstruction [1]. It is hypothesized to affect 4 percent of the adult population, 82 percent of who are women [2]. Patients with gastroparesis can experience symptoms of early protracted satiety, nausea, vomiting, bloating and abdominal pain. Gastroparesis occurs in neuromuscular or infiltrating disorders that interfere with gastric transit. One important association with gastroparesis is diabetes. In patients with diabetes, the incidence of gastroparesis is 40 percent in type 1 patients and 30 percent in type 2 patients, respectively [2,3]. In one study, gastrointestinal disorders were present despite glycemic control and were found in association with depression [4]. Several studies have investigated the link between glycemic control and the development of gastroparesis, but the results have often been inconsistent [5]. Although gastroparesis seems to develop several years after the diagnosis of diabetes and earlier in patients with poor glycemic control, the gastrointestinal symptoms observed are similar in patients who are non-diabetic. Gastric emptying scintigraphy is the gold standard for diagnosis of gastroparesis and consists of measuring the transit time of a radiolabeled solid meal. If greater than 10 percent of the meal remains four hours after ingestion, then there is gastroparesis [2]. Studies such as a wireless motility capsule and stable isotope breath test may also be used for diagnosis, and employ measurement of the capsule passage from the stomach to the duodenum or the breakdown of labeled medium-chain triglycerides, respectively [3]. Gastrointestinal transit may also be assessed simply by using serial abdominal radiographs and measuring radiolabeled stool output [6].

Treatment of gastroparesis can be very challenging for those patients who have it. Therapy usually begins with correction of underlying electrolyte abnormalities and enhanced glucose control in diabetics, as it has been shown that hyperglycemia slows gastric emptying [7]. Medication management usually includes an anti-emetic agent for symptom control, in combination with a pro-kinetic agent to stimulate movement. The underlying pathophysiology involves enhancing neurotransmitters, primarily dopamine, motilin and serotonin [2]. Common agents that work on the dopaminergic neurotransmitters include phenothiazine, metoclopramide, and domperidone. Unfortunately, there are numerous side effects including drowsiness, extrapyramidal symptoms, xerostomia and rash. Erythromycin and low dose tricyclic antidepressants have also been employed with some success, but erythromycin commonly causes diarrhea and tricyclics may cause anti-cholinergic effects that enhance symptoms. More invasive management strategies include implantation of a gastric electric stimulator, which has been shown to improve nausea and vomiting within six weeks of therapy, and botulinum toxin injection is continually being investigated for efficacy [8,9]. Despite these options, there remains a clear need to look at other potential medications for gastroparesis.

## Case presentation

An 87-year-old African-American woman who was a nursing home resident with a history of diabetes mellitus type 2 came to the attention of medical staff because of continued high post-feed residuals from her tube feedings. She was aphasic and could not give a direct history to the staff. Her pertinent medical history included diabetes diagnosed nine years previously, subarachnoid hemorrhage leading to her aphasia, hemiplegia, seizures and dysphagia requiring percutaneous gastric feeds. She was completely dependent upon the tube feedings for nutritional and hydration support. She was noted to have recurrent monthly episodes of aspiration pneumonia, abdominal bloating, vomiting and large tube-feed residuals consistent with a diagnosis of underlying gastroparesis. With her ongoing decline, our patient was in a palliative state, desiring comfort measures with a limitation on testing. As a result, gastric emptying studies were not undertaken. On examination, she was aphasic with a percutaneous gastric tube in place and residuals of greater than 150mL. Her medications included allopurinol, buspirone, calcium and vitamin D, citalopram, insulin, levetiracetam, furosemide, levothyroxine, metoprolol, mexiletine, amlodipine, omeprazole, gabapentin and a prednisone taper for bullous pemphigoid. Laboratory studies showed no abnormalities and an abdominal X-ray showed no evidence of mechanical obstruction. She was started on a course of metoclopramide 30mg for pro-kinetic effect and prochlorperazine 25mg daily for nausea. Both medications provided temporary improvement in nausea and vomiting and modest reductions in her residuals.

Her nursing home course was complicated by continued episodes of recurrent aspiration pneumonia requiring hospitalization and oxygen supplementation. The doses of her anti-emetic and pro-kinetic agents were adjusted and her tube feeds were slowed significantly. There were considerable risks to our patient as the staff continued to slow the tube feeds, which placed her at risk for low nutritional intake. Despite the changes, within a couple months, her increased post-void residuals returned. Her clinical picture and family wishes precluded invasive therapies such as a gastric electric stimulator and botulinum injections. Tricyclics and erythromycin were not considered ideal with her underlying mental and neurologic disorders and medication regimen. After review of the literature for non-invasive therapies to manage recalcitrant gastroparesis, two reports of success with mirtazapine were found. One case report involved a patient with post-gastropexy-related recalcitrant gastroparesis that promptly resolved with mirtazapine [10]. In another report, a 27-year-old woman with diabetes type 1 and gastroparesis recalcitrant to seven months of therapy with all known medication regimen and botulinum toxin injections, had resolution of her symptoms within one week of therapy with 15mg of mirtazapine [11].

Our patient’s residual volume decreased from greater than 170mL to less than 70mL and she had no aspiration pneumonia for months prior to her death. Efficacy was noted at a threshold dose of 15mg nightly.

## Discussion

Gastroparesis can be a challenging and life-threatening complication of diabetes mellitus. In our patient, gastroparesis contributed to recurrent aspiration pneumonias in a debilitated nursing home resident and was likely secondary to long-standing diabetes as there is an incidence of up to 50 percent in such patients [12]. We used the first-line therapies of metoclopramide and prochlorperazine at varying doses to no lasting avail. Our patient was not an ideal candidate for erythromycin, tricyclics or invasive management. Mirtazapine provided improvement in her post-feeding residuals and reduced her aspiration events. Mirtazapine is a serotonergic 5-hydroxytryptamine 2/3 (5HT2/3) receptor antagonist and noradrenergic antidepressant [13], which has been used off-label for management of gastroparesis in the cases as described. Its exact mechanism of action for gastroparesis is unclear but thought related to serotonergic and noradrenergic effects. Its effects on relieving gastroparesis are thought to be related to potent 5-HT3 antagonism and has been reported effective in relieving post-operative and recalcitrant severe gastroparesis [10,11], however, there is very little evidence to support or standardize this off-label use. There have been no randomized control trials for further investigation although mirtazapine is an attractive alternative non-invasive regimen with minimal side effects and favorable efficacy. It also has the additional benefit of being an antidepressant. It is worthwhile to employ this therapy when other primary therapies have failed. Mirtazapine is especially helpful for patients in whom invasive interventions are impractical, including patients who are older, desire non-invasive care or have multiple comorbidities that preclude procedures. The use of mirtazapine has never been reported with regards to reduced tube-feed residuals.

## Conclusion

This report presents the case of an older African-American woman with long-standing diabetes who developed gastroparesis after a debilitating cerebrovascular accident. Her symptoms were temporarily managed with conventional anti-emetic and pro-kinetic therapies but later became persistent until the initiation of mirtazapine. In patients with gastroparesis that is recalcitrant to first-line therapies, or where invasive therapies are impractical, off-label use of mirtazapine may provide non-invasive management of persistent gastroparetic symptoms. The mechanism of its effect on gastroparesis has not been clearly elucidated or studied, and there has been only one non-surgical case previously reported in the literature.

## Consent

Written consent was obtained from the patient’s next of kin and is available for review by the Editor-in-Chief of this journal upon request.

## Competing interests

The authors declare that they have no competing interests.

## Authors’ contributions

JG: managed our patient, acquired the data and composed the manuscript. PT: supervised management decisions, reviewed and contributed to the compilation of the manuscript. Both authors read and approved the final manuscript.
